# Anatomical dissection influences emotions of podiatry students

**DOI:** 10.1002/jfa2.70027

**Published:** 2025-01-13

**Authors:** Alicia Mohedano‐Moriano, Carmen Romo‐Barrientos, Alicia Flores‐Cuadrado, Isabel Ubeda‐Bañon, Jaime Gonzalez‐Gonzalez, Maria Teresa Gil Ruiz, Daniel Saiz‐Sanchez, Veronica Astillero‐Lopez, Felix Marcos‐Tejedor, Alino Martinez‐Marcos, Antonio Viñuela, Juan Jose Criado‐Alvarez

**Affiliations:** ^1^ Department of Medical Sciences Faculty of Health Sciences Research Group Technological Innovation Applied to Health (ITAS) University of Castilla‐La Mancha Talavera de la Reina Toledo Spain; ^2^ Teaching Innovation Group Multidisciplinary Integration and Anatomical Dissemination University of Castilla‐La Mancha Ciudad Real Spain; ^3^ Integrated Attention Management of Talavera de la Reina Castilla‐La Mancha Health Service (SESCAM) Talavera de la Reina Toledo Spain; ^4^ Department of Medical Sciences Ciudad Real Medical School, IB‐UCLM IDISCAM, University of Castilla‐La Mancha, Ciudad Real Ciudad Real Spain; ^5^ Department of Nursing, Physiotherapy and Occupational Therapy, Faculty of Health Sciences Research Group Technological Innovation Applied to Health (ITAS), University of Castilla‐La Mancha Talavera de la Reina Toledo Spain; ^6^ Institute of Health Sciences of Castilla‐La Mancha Talavera de la Reina Toledo Spain

**Keywords:** anatomy, anxiety, dissection, education, prosection

## Abstract

**Background:**

Dissection and examination of prosected cadavers is a tool for teaching anatomy. However, this experience can provoke anxiety and stress among students. This study aims to understand the attitudes, reactions, fears, and anxiety states of podiatry students before their first dissection in addition to evaluate its usefulness as an educational tool for academic training in anatomy.

**Methods:**

A cross‐sectional study was carried out before and after the dissection room visit of first‐year podiatry students. They were given several questionnaires: State‐Trait Anxiety Inventory questionnaires (STAI‐state anxiety and STAI‐trait anxiety) and two anonymous questionnaires.

**Results:**

Levels of total emotional anxiety (STAI‐state anxiety) decreased significantly (*p* < 0.05) from 16.9 points before practice to 10.9 points after practice. In terms of gender, significant differences (*p* < 0.05) were observed in anxiety levels before and after practice. However, female students had significantly (*p* < 0.05) higher pre‐practice levels of STAI‐state anxiety than male students.

**Conclusions:**

Although 100% of students (3.98 ± 0.149, over 4) expressed satisfaction with the practical's dissection and considered that these contributed significantly to the consolidation of their anatomical knowledge, the experience generated emotional responses that need to be addressed. Higher levels of anxiety were observed among female students, highlighting the need to implement effective coping mechanisms to mitigate emotional reactions, with special emphasis on this population.

## 1 INTRODUCTION

The subject of human anatomy is a component of the curriculum of podiatry degree [1, 2], and it is found in block 1 of the 8 blocks that make up the curriculum (1. Structure and function of the human body, 2. General Pathology and Microbiology, 3. Pharmacology and Pharmacological Therapy, 4. Public Health and Psychosocial Sciences, 5. Biomechanics and General Podiatry, 6. Podiatric Pathology, Orthopedic Treatments and Physicists, 7. Chiropody, Podiatric Surgery, and 8. Integrated Clinical Practicum) [3]. The subjects in Block 1 include Human Anatomy and Human Anatomy of the Lower Limb, which are a basic pillar for future subjects such as pathology, surgery, or physical podiatry.

Dissection is one of the tools used for teaching human anatomy and is considered one of the best learning methods [4, 5, 6, 7]. Practical dissection or prosection sessions do not form part of most health sciences curricula in Spain, except medicine [8, 9]. The practical content of this subject in many podiatry degrees is delivered in the gross anatomy laboratory through palpation, anatomical models, and technological resources. Dissection or prosection practical sessions tend to not exist, and are not mandatory, because gross anatomy laboratories with human cadavers are difficult to achieve and to maintain [10]. This does not mean, however, that dissection was not considered to be a highly effective and useful learning tool for learning anatomy. It includes benefits that cannot be achieved through other methods [11, 12]. Dissection helps to improve spatial reasoning and the understanding of anatomical relationships [11, 13, 14, 15] while students work as a group in the dissection room and develop mental habits for clinical practice [11, 16]. It is a useful tool to study human variability [17]. It also promotes the integration of affective and cognitive skills and encourages self‐reflection on issues such as death and illness [18]. All of these are essential skills for the podiatrist's professional practice. However, due to the experience of dealing with death, touching and seeing a cadaver for the first time, or for ethical and religious reasons, this experience can generate stress in some students [7, 19, 20, 21].

Previous studies, such as those by Tanner [22], report that stress and anxiety can hinder learning. Numerous studies have shown that this state of anxiety and these negative feelings prior to the first dissection decrease significantly and are not maintained over time [20, 23]. Nevertheless, anatomical knowledge is fundamental for podiatric qualifications and is essential for subsequent surgical practice. Poor anatomical proficiency can lead to errors in surgical procedures [24]. Furthermore, if teaching focuses on normality, neglecting anatomical variability, the level of errors may further increase [17]. This is compounded by the lack of in‐depth learning in this subject [25, 26], coupled with the fact that it is taught in the early years of health science degrees. There is also limited vertical integration with later years of the curriculum. Finally, in the last decade, there has been a reduction in the content of this subject [8], and in particular, a reduction of practical hours in the dissection room [6].

Therefore, the aim of this study is to try to understand the state of anxiety before and after the first dissection/prosection of a cadaver and the attitudes, reactions, and fears detected in first‐year podiatry students. A further aim is to study students' evaluation of these practical sessions. This information is of great interest with a view to incorporating these practical sessions into the podiatry curriculum, where dissection is not currently included in the anatomy program. Finally, input from both students and educators is crucial to designing a more successful anatomy curriculum [13].

## 2 MATERIALS AND METHODS

### 2.1 Anatomy in the podiatry curriculum

All degree programs in podiatry comprise 240 ECTS (European Credit Transfer System) and have a duration of four academic years. In the Talavera de la Reina podiatry program, gross anatomy courses are conducted in the first year. During the first year, two subjects are taught: “Human Anatomy” and “Human Anatomy of the Lower Limb”. Each subject has four theoretical and two practical ECTS (60 contact hours in total). One ECTS is equivalent to 25–30 h of total work for students. 10 h are allocated to face‐to‐face classes (theoretical classes and gross anatomy laboratory equipped with ossuaries, anatomical models, and 3D atlases), and the rest of the hours correspond to study time and work by the student.

### 2.2 Sample

This is a descriptive pre‐/post‐ study conducted in the 2021/2022 academic year with 45 first‐year students enrolled in the course on Anatomy of the Lower Limb (6 ECTS, of which 4 ECTS are theoretical, with 40 teaching hours, and 2 ECTS are practical, with 20 h of in‐person practical sessions), as part of the degree in podiatry offered at the Faculty of Health Sciences at the University of Castilla‐La Mancha (UCLM). The students on this course worked on cadaver prosections/dissections. Student participation in this activity was voluntary. All participants used regulatory protective equipment in the sessions and were also informed about the cadaver donation program accessible on the website of the Ciudad Real Medical School (UCLM, 2022). Furthermore, it was the first time this teaching tool had been used for students enrolled in this course to learn anatomy. The sessions lasted 6 h, during the first 3 h of which the following content was addressed by means of prosection: visual recognition of the region to be studied (gluteal region; anterior, medial, and posterior thigh; posterior, anterior, and lateral leg; and back and sole of the foot), location of structures by plans, and palpation and recognition of the relationship between the different structures. After a break, the second 3‐h session corresponded to dissecting the foot's sole. The foot dissection was performed in small groups of 3 students.

### 2.3 Procedures and instruments

Twenty minutes before entering the dissection room, students answered two questionnaires: an anonymous pre‐questionnaire (created for this study) and the State–Trait Anxiety Inventory (STAI) [27]. The anonymous questionnaire (with 12 items) was used to understand feelings and emotions before participating in the dissection. This questionnaire is a non‐validated scale based on the model designed by Miguel Pérez [28] used in other articles [9, 29]. The STAI is a self‐administered questionnaire, validated in a Spanish population, and designed to measure anxiety in healthy adults. The STAI has a Cronbach's alpha of 0.93 for STAI‐AT and 0.92 for STAI‐AS [24]. It consists of two scales measuring state anxiety (STAI‐AS) and trait anxiety (STAI‐AT). The STAI‐AT measures an individual's baseline level, while the STAI‐AS assesses how a person feels in a specific stressful situation. Both questionnaires consist of 20 questions that are answered on a 4‐point Likert‐type scale, with the following distribution: 0 = not at all, 1 = somewhat, 2 = quite a lot, and 3 = very much. To avoid interference, participants were not informed beforehand that they would have to complete the questionnaires (STAI and an anonymous post‐questionnaire) at the end of the practical session. The post‐questionnaire had 15 questions of which 9 were common to the pre‐questionnaire. This questionnaire is used to evaluate feelings and emotions together with the degree of satisfaction with the activity and the evaluation of the practical sessions.

### 2.4 Statistical analysis

Descriptive and inferential statistical analyses were performed. Different parameters were used according to the scales of the variables. A *t*‐test was conducted for the paired variables and a chi‐square test for the comparison and dichotomous variables. A confidence level of 5% was established. SPSS 28.0 for Windows was used for data analysis.

### 2.5 Ethics

The students were informed of the general objectives of the study, and their anonymity was guaranteed. Informed consent was requested, which students were told that they could revoke at any time. In addition, this study was approved by the Talavera de Reina (Toledo) Clinical Research Ethics Committee (File 23/2017).

## 3 RESULTS

A total of 45 students participated (100% of the group, of which 72.5% were females); their mean age was 19.7 ± 2.39 years (median: 19; range: 18–30).

Regarding the STAI questionnaire, in the first phase (before entering the dissection room), the STAI‐AT score was 21.3 ± 10.04 points (median: 21; range: 21–28).

In the total STAI‐AS questionnaire, the values decreased significantly (*p* < 0.05) from the first phase with a score of 16.9 ± 10.02 points to the second phase (immediately after the end of the practical session, when leaving the dissection room) with a score of 10.9 ± 7.55 points.

**FIGURE 1:**
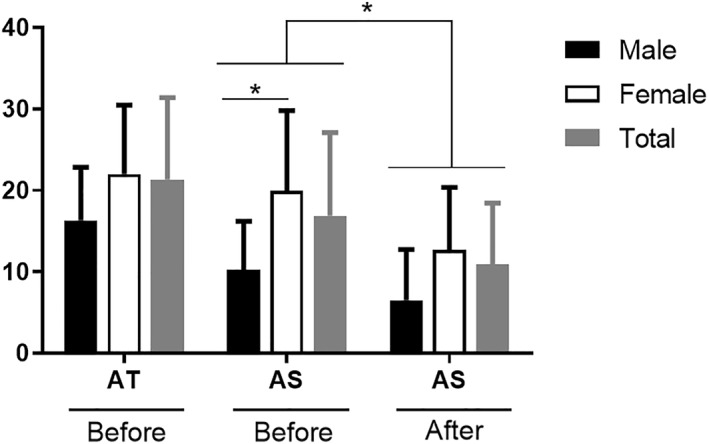
STAI‐trait anxiety (AT) and STAI‐state anxiety (AS) results by gender and during practice. Asterisk means significant differences (*p* < 0.05).

When analyzing the data according to gender, significant differences were observed (*p* < 0.05). Females had higher scores (STAI‐AS, 19.9 ± 9.91 points) than males (STAI‐AS 10.2 ± 6.00) in the first phase. Although in the second phase the scores decreased, the differences between genders remained significant, with women's STAI‐AS scores 12.7 ± 7.68 points and men's STAI‐AS scores 6.5 ± 6.26 points (Figure 1).

**FIGURE 2:**
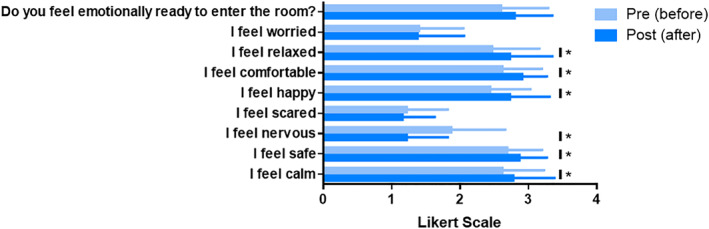
Students’ feelings during practice. Light blue, pre‐ (before entering the dissection room) and dark blue, post (after entering the dissection room). Asterisk means significant differences (*p* < 0.05).

The results obtained from the pre‐questionnaire during the first phase (before entering the dissection room) were as follows. In general, 40% (mean: 1.40 ± 0.49) of the students had never seen a cadaver (n: 18; of which 13 were female), with the students reporting they felt “calm” (mean: 2.62 ± 0.61) and “safe” (mean: 2,69 ± 0.51) more than “nervous” (mean: 1.87 ± 0.79), “scared” (mean: 1.22 ± 0.6), or “worried” (mean: 1.4 ± 0.65). A total of 48.9% felt “happy” (mean: 2.44 ± 0.59), in addition to feeling “relaxed” and “comfortable” (mean: 2.47 ± 0.69 and 2.62 ± 0.58), respectively. Additionally, 71.1% (mean: 2.60 ± 0.69) felt emotionally prepared to enter the dissection room (see Figure 2, Supplementary Table 1).

Regarding what they found most unpleasant before entering the dissection room, the students replied that it was “seeing the face of the cadaver” (55.6%, mean: 1.56 ± 0.5) and the “smell” (53.3%, mean: 1.53 ± 0.5), in comparison with “touching the cadaver” (24.4%, mean: 1.24 ± 0.43). Regarding the thoughts generated by the dissection, the students reported great “curiosity” (95.6%, mean: 1.96 ± 0.21), followed by “uncertainty” (23%, mean: 1.96 ± 0.21) (see Table 1).

**TABLE 1:** Student thoughts before entering the dissection room.

		Yes		No	
		*N*	%	*N*	%
The thought of dissection produces	Anxiety	2	4.4	43	95.6
	Disgust	5	11.1	40	88.9
	Curiosity	42	95.6	2	4.4
	Uncertainty	22	48.9	23	51.1
	Fear	2	4.4	43	95.6
What is the unpleasant part of the dissection room?	See the face of the corpse	25	55.6	20	44.4
	The smell of the dissection	24	53.3	21	46.7
	Touch the corpse	11	24.4	34	75.6

After the practical session in the dissection room, the results obtained from the post‐questionnaire showed that the students reported feeling more “safe” (mean: 2.87 ± 0.40), “relaxed” (mean: 2.73 ± 0.62), “happy” (mean: 2.73 ± 0.58), and “comfortable” (mean: 2.91 ± 0.36) with statistically significant differences (*p* < 0.05). In addition, they felt less “nervous” and “scared” (mean: 1.22 ± 0.60 and mean: 1.16 ± 0.47, respectively), with statistically significant differences (*p* < 0.05). There was a slight increase in feeling “worried” (mean: 1.38 ± 0.68) and being more prepared to enter the dissection room (mean: 2.80 ± 0.55), but without significant differences (see Figure 2, Supplementary Table 1).

After finishing the practical session, 55.6% (mean: 1.56 ± 0.50) reported “thinking about life or death in the dissection room”, and 22.2% (mean: 1.22 ± 0.42) said they had been “afraid of losing control in the room”, while only 45.2% (mean: 1.45 ± 0.50) said they would “donate their body to science”.

In feelings during the practical session, the female students in the first phase (pre‐, before entering the dissection room) felt less “calm” and “happy” and more “worried” and “nervous” than the male students, but without significant differences. However, significant differences were found for the same gender between first phase (pre‐) and second phases (post, immediately after finishing the practical session, upon leaving the dissection room) for the items of “nervous” and “comfortable”. These differences are the same in the case of female students, although two further items, namely, “happy” and “relaxed”, also showed significant differences (Supplementary Table 2).

Finally, the students' score for satisfaction with their first dissection session was 9.6 ± 0.52 points (median: 10; range: 8–10), with 1 and 44 students being “satisfied” or “very satisfied” (2.2% and 97.8%, first phase, and mean: 3.98 ± 0.15), respectively. All the students recommended these practical sessions be included in subsequent courses. The general evaluation of the practical sessions included in the anatomy course during the academic year was 3.05 ± 0.78 out of 4 points. Of these practical sessions, “dissection” scored higher (3.20 ± 0.81) than other practical resources, such as clinical cases or radiological identification of anatomical structures. In addition, 87.2% of the students consider this tool useful for their learning.

## 4 DISCUSSION

### 4.1 Dissection practices

Anatomical knowledge is essential for health sciences degrees such as podiatry, being essential for ulterior surgical practice. Poor anatomical proficiency can lead to errors in surgical procedures [17]. In addition, whether teaching is focused on normality, neglecting anatomical variability, the level of errors can be further increased [25]. This is worsened by the lack of deep learning in this subject [30, 31] together with the fact that it being taught in the first years of the health sciences degrees. There is also a limited vertical integration with subsequent years of the curricula. Finally, in the last decade a content reduction of this subject has occurred [8], and in particular, a reduction in practical hours in the dissection room [6].

Dissection or prosection practical sessions are frequent in medical education, but not always included in podiatry due to the high cost of human‐donned bodies and gross anatomy laboratories [10]. Dissections and prosections are a fundamental for anatomical learning, not only for understanding of anatomical structures but also their spatial relationships. Also, it fosters the integration of affective and cognitive skills, while performing group work in the dissection room and develops mental habits for clinical practice [11, 18]. In addition, it is a useful tool for studying human variability [25] and encourages self‐reflection on issues such as death and disease [8, 30]. These are all essential skills for the professional practice of podiatrist.

For most anatomical teachers, dissection is irreplaceable for anatomy learning [6]. Students also consider it essential for their training [7, 31]. They emphasize its utility for understanding the human body beyond theoretical lessons [32]. Many reports support student preference comparing dissection as web‐based approaches [33, 34, 35]. In fact, our students preferred dissection (3.20 out of 4 points) to clinical cases or 2D radiological images, like other studies related to health sciences that considered dissection a useful tool (94%) compared to other traditional tools (anatomical models, books, images, or Internet) [36].

Podiatry students rated this practical dissection and prosection session with an average of 9.6 ± 0.52 points out of 10. These data are consistent with other studies in the field of health sciences, where scores for dissection sessions reach similar values [37, 38]. Students consider these practical sessions to be a useful tool to consolidate anatomical knowledge, with our results being similar to those of Babinski, who reported that 81% of students considered dissection useful for their learning [39]. In addition, other researchers confirm the usefulness of this methodology in consolidating learning [35].

Dissection on human cadavers has multiple learning advantages: proactive approach, spatial relationships, hand skill, teamwork and also prepares students for clinical practice, and facing stress and death [7, 35, 40, 41, 42]. In the last decade, dissection has been positive and negatively criticized. Detractors argue high cost, toxic environment and ethical issues as well necessity of qualified personnel [43, 44, 45, 46].

In the last years, emerging technologies such as “anatomage” table, virtual, and augmented reality and 3D printing have replaced dissection [47]. Some studies report that 3D models and gamification have positive effects against dissection because they are less stressful [48, 49]. Other studies found that augmented reality was not significantly different to traditional 2D or 3D models [50, 51, 52] as is the case for computer‐based learning [53, 54]. “Anatomage” Table is considered a powerful tool for studying internal structures [55, 56], but it has been demonstrated that student results comparing dissection or 3D visualization are similar [57, 58].

New methodologies are important and useful in the teaching of anatomy, but not to replace traditional methodologies but to complement them [59, 60].

### 4.2 Emotions and feelings

Although these practical sessions are positively evaluated, as seen above, they can be the cause of stressful experiences [4, 9, 61, 62, 63]. The results of the STAI‐AS decreased from the first phase (pre‐) to the second phase (post) with the differences being statistically significant (*p* < 0.05), similar to data reported for other degrees in health sciences [19, 61, 62, 64].

According to gender, female students in the first phase (pre‐) were more anxious (STAI‐AS) than their male counterparts, with significant differences (*p* < 0.05). These data are interesting because the learning of female students may be affected, as studies have shown that anxiety can negatively intervene in learning and decision‐making [22, 65, 66]. In addition, from a numerical perspective, the female students were less emotionally prepared to enter the room and felt more “scared”, “worried”, and “nervous” than the males in the first phase (pre‐), although these differences were nonsignificant. These data coincide with those from other studies suggesting that female students experience higher levels of negative emotions than males when dealing with dissection [67, 68, 69, 70]. However, in our case, once the first dissection/prosection session was finished, the values by genders were equalized while other studies have suggested the differences in values are maintained over further sessions [70], although they finally draw equal after time [71].

Regarding negative emotional responses, the students considered “the smell of the dissection room”, “seeing the face of the cadaver”, and “touching the body” to be unpleasant, (53.3%; 55.6%, and 24.4%, respectively). These data coincide with those for other health sciences degree, where between 44.4% and 70% of participants described the smell as unpleasant [37, 70, 71, 72, 73, 74]. In a study with podiatry students, 49.2% of the participants referred to “the smell of the dissection room” as a cause of distress [75]. Regarding “seeing the face of the cadaver” or “touching a body”, our students reported feeling uncomfortable, as in other studies [35, 76, 77]. Indeed, these feelings make our students prefer alternative methodologies [48].

As regards the positive emotional responses, 95.6% of the students were “curious” about this practical session, being one of the highest values reported, compared to findings for other health science degrees, which are in a range of 80%–89% [62, 64, 78]. In this sense, it is worth noting that curiosity is a fundamental element in academic performance [79, 80]. Regarding the emotions of “calm”, “safe”, “happy”, “relaxed”, “emotional”, and being “more prepared to enter the dissecting room”, these tended to increase at the end of the practical sessions.

Finally, one limitation of the present study was the small sample size (45 students were enrolled in the podiatry degree). Furthermore, this was the first year that dissections/prosections were used as a complementary methodology for teaching anatomy. Therefore, no previous results were available to compare current findings.

## 5 CONCLUSIONS

The basis for future professional practice of podiatrists should be achieved with the correct learning of the anatomy of the lower limb. Students find dissection/prosection a useful learning tool, and even prefer it to other alternatives such as clinical cases or radiological images.

However, it should be noticed that these practical sessions can affect them emotionally. Anxiety and discomfort, especially in female students, should be prevented by positive coping techniques to reduce the negative emotional impact of the first exposures to cadavers.

Finally, authors propose that dissection and prosection sessions should be mandatory as part of the lower limb human anatomy curriculum. The study underlines the importance of dissection and prosection in the training of future podiatrists, because it provides a direct anatomical experience that significantly improves their understanding of the lower limb.

## AUTHOR CONTRIBUTIONS

Conceptualization, A.M.M1., C.R.B. and J.J.C.A.; methodology, A.M.M1, C.R.B., A.F.C., I.U.B. and J.J.C.A.; software, A.V. and J.J.C.A.; formal analysis, C.R.B. and A.F.C.; investigation, J.G.G., M.T.G.R., D.S.S., V.A.L., F.M.T. and A.M.M2.; resources, A.V.; writing—original draft preparation, A.M.M1. and J.J.C.A.; writing—review and editing, C.R.B., A.F.C., I.U.B., A.M.M2 and J.J.C.A.; visualization, A.V.; supervision, A.M.M1 and A.F.C.; All authors have read and agreed to the published version of the manuscript.

## CONFLICT OF INTEREST STATEMENT

The authors declare no conflicts of interest.

## ETHICS STATEMENT

This study was approved by the Talavera de Reina (Toledo) Clinical Research Ethics Committee (File 23/2017).

## Supporting information

Supplementary Material

## Data Availability

The data that support the findings of this study are available in the supplementary material of this article.
