# Supplementary material for: Anatomical dissection influences emotions of podiatry students
Source: J Foot Ankle Res. 2025 Jan 13;18(1):e70027. doi: 10.1002/jfa2.70027 (PMC11727409; doi:10.1002/jfa2.70027)
Supplement: Supplementary file 1 — Supplementary Material [file JFA2-18-e70027-s001.docx]

**Supplementary Table 1.**

**Students' feelings during practice**

|  | **Pre (before)** | | | | | | | | | **Post (after)** | | | | | | | | |  | |
| --- | --- | --- | --- | --- | --- | --- | --- | --- | --- | --- | --- | --- | --- | --- | --- | --- | --- | --- | --- | --- |
|  | **Yes** | | | **Indifferent** | | | **No** | | | **Yes** | | | **Indifferent** | | | **No** | | | ***P*-value** | |
|  | **n** | **%** | **n** | | **%** | **n** | | **%** | **N** | | **%** | **n** | | **%** | **n** | | **%** |  | |  |
| **I feel calm** | 31 | 68.9 | 11 | | 24.4 | 3 | | 6.7 | 39 | | 86.7 | 2 | | 4.4 | 4 | | 8.9 | p>0.05* | |  |
| **I feel safe** | 32 | 71.1 | 12 | | 26.7 | 1 | | 2.2 | 40 | | 88.9 | 4 | | 8.9 | 1 | | 2.2 | p<0.05* | |  |
| **I feel nervous** | 11 | 24.4 | 17 | | 37.8 | 17 | | 37.8 | 4 | | 8.9 | 2 | | 4.4 | 39 | | 86.7 | p<0.05* | |  |
| **I feel scared** | 4 | 8.9 | 2 | | 4.4 | 39 | | 86.7 | 2 | | 4.4 | 3 | | 6.7 | 40 | | 88.9 | p>0.05 | |  |
| **I feel happy** | 22 | 48.9 | 21 | | 46,7 | 2 | | 4.4 | 36 | | 80.0 | 6 | | 13.3 | 3 | | 6.7 | p<0.05* | |  |
| **I feel comfortable** | 30 | 66.7 | 13 | | 28.9 | 2 | | 4.4 | 42 | | 93.3 | 2 | | 4.4 | 1 | | 2.2 | p<0.05* | |  |
| **I feel relaxed** | 26 | 57.8 | 14 | | 31.1 | 5 | | 11.1 | 37 | | 82.2 | 4 | | 8.9 | 4 | | 8.9 | p<0.05* | |  |
| **I feel worried** | 4 | 8.9 | 10 | | 22.2 | 31 | | 68.9 | 5 | | 11.1 | 7 | | 15.6 | 33 | | 73.3 | p>0.05 | |  |
| **Do you feel emotionally ready to enter the room?** | 32 | 71.1 | 8 | | 17.8 | 5 | | 11.1 | 39 | | 86.7 | 3 | | 6.7 | 3 | | 6.7 | p>0.05 | |  |

**Supplementary Table 2**

**Students' feelings during practice by gender**

| **Students' feelings during practice by gender** | **Pre (before)** | | | **Post (after)** | | |  |
| --- | --- | --- | --- | --- | --- | --- | --- |
|  | **%** | | | **%** | | |  |
|  | **Yes** | **Indifferent** | **no** | **Yes** | **Indifferent** | **No** | **P-value** |
| I feel calm male | 91.7 | 8.3 | 0 | 91.7 | 0 | 8.3 | p>0.05 |
| I feel calm female | 58.6 | 34.5 | 6.9 | 82.8 | 6.9 | 10.3 | p>0.05 |
| I feel safe male | 75 | 25 | 0 | 91.7 | 8.3 | 0 | p>0.05 |
| I feel safe female | 69 | 31 | 0 | 86.2 | 10.3 | 3.4 | p>0.05 |
| I feel nervous male | 8.3 | 58.3 | 33.3 | 8.3 | 0 | 91.7 | **p<0.05** |
| I feel nervous female | 31 | 31 | 37.9 | 10.3 | 6.9 | 82.8 | **p<0.05** |
| I feel scared male | 0 | 8.3 | 91.7 | 0 | 0 | 100 | p>0.05 |
| I feel scared female | 10.3 | 3.4 | 86.2 | 6.9 | 10.3 | 82.8 | p>0.05 |
| I feel happy male | 66.7 | 33.3 | 0 | 83.3 | 8.3 | 8.3 | p>0.05 |
| I feel happy female | 37.9 | 55.2 | 6.9 | 75.9 | 17.2 | 6.9 | **p<0.05** |
| I feel comfortable male | 66.7 | 33.3 | 0 | 100 | 0 | 0 | **p<0.05** |
| I feel comfortable female | 65.5 | 27.6 | 6.9 | 89.7 | 6.9 | 3.4 | **p<0.05** |
| I feel relaxed male | 66.7 | 25 | 8.3 | 83.3 | 0 | 16.7 | p>0.05 |
| I feel relaxed female | 51.7 | 37.9 | 10.3 | 79.3 | 13.8 | 6.9 | **p<0.05** |
| I feel worried male | 0 | 16.7 | 83.3 | 16,7 | 0 | 83,3 | p>0.05 |
| I feel worried female | 10,3 | 27,6 | 62,1 | 6.9 | 24.1 | 69 | p>0.05 |
| Do you feel emotionally ready to enter the room? male | 83.3 | 8.3 | 8.3 | 83.3 | 8.3 | 8.3 | p>0.05 |
| Do you feel emotionally ready to enter the room? female | 69 | 20.7 | 10.3 | 86.2 | 6.9 | 6.9 | p>0.05 |
| p-value | p>0.05 for the 9 items between female and male | | | p>0.05 for the 9 items between female and male | | |  |
